# The Relationship of Within-Host Multiplication and Virulence in a Plant-Virus System

**DOI:** 10.1371/journal.pone.0000786

**Published:** 2007-08-29

**Authors:** Israel Pagán, Carlos Alonso-Blanco, Fernando García-Arenal

**Affiliations:** 1 Departamento de Biotecnología and Centro de Biotecnología y Genómica de Plantas, Universidad Politécnica de Madrid, Madrid, Spain; 2 Departamento de Genética Molecular de Plantas, Centro Nacional de Biotecnología, Consejo Superior de Investigaciones Científicas, Universidad Autónoma, Cantoblanco, Madrid, Spain; Oxford University, United Kingdom

## Abstract

**Background:**

Virulence does not represent any obvious advantage to parasites. Most models of virulence evolution assume that virulence is an unavoidable consequence of within-host multiplication of parasites, resulting in trade-offs between within-host multiplication and between-host transmission fitness components. Experimental support for the central assumption of this hypothesis, *i.e.*, for a positive correlation between within-host multiplication rates and virulence, is limited for plant-parasite systems.

**Methodology/Principal Findings:**

We have addressed this issue in the system *Arabidopsis thaliana*-*Cucumber mosaic virus* (CMV). Virus multiplication and the effect of infection on plant growth and on viable seed production were quantified for 21 *Arabidopsis* wild genotypes infected by 3 CMV isolates. The effect of infection on plant growth and seed production depended of plant architecture and length of postembryonic life cycle, two genetically-determined traits, as well as on the time of infection in the plant's life cycle. A relationship between virus multiplication and virulence was not a general feature of this host-parasite system. This could be explained by tolerance mechanisms determined by the host genotype and operating differently on two components of plant fitness, biomass production and resource allocation to seeds. However, a positive relationship between virus multiplication and virulence was detected for some accessions with short life cycle and high seed weight to biomass ratio, which show lower levels of tolerance to infection.

**Conclusions/Significance:**

These results show that genotype-specific tolerance mechanisms may lead to the absence of a clear relationship between parasite multiplication and virulence. Furthermore, a positive correlation between parasite multiplication and virulence may occur only in some genotypes and/or environmental conditions for a given host-parasite system. Thus, our results challenge the general validity of the trade-off hypothesis for virulence evolution, and stress the need of considering the effect of both the host and parasite genotypes in analyses of host-parasite interactions.

## Introduction

Parasites are an important fraction of living organisms and influence all levels of biological organisation. Explaining virulence, defined as the deleterious effects of parasites on their hosts [1] is fundamental to understand the life history of parasites. Understanding virulence may be also of socio-economic relevance due to the important impact of infectious diseases on human, animal and plant welfare [1]–[4]. Because virulence does not represent any clear advantage for parasites, which depend on their hosts for survival and fitness, it is not obvious why parasites harm their hosts. A commonly accepted hypothesis is that virulence is an unavoidable consequence of parasite multiplication within the infected host [5]. Consequently, virulence will result in trade-offs between different components of the parasite's fitness. Since the seminal work of Anderson and May [6], formal analyses of the evolution of virulence have been based on the assumption of such a trade-off between within-host multiplication and the between-host transmission components, *i.e.*, on the trade-off hypothesis. The trade-off hypothesis has resulted in a large body of theoretical work aimed at understanding different aspects of pathogen evolution and of host-pathogen co-evolution [7]–[9]. However, experimental analyses of the trade-off hypothesis are comparatively scarce and apparently contradictory. The general validity of the trade-off hypothesis and of its central assumption of a positive correlation between parasite multiplication and virulence has been questioned for more than a decade. Other alternative hypotheses have been proposed to explain virulence, which take into account the diversity of parasite's life cycles and the specificities of the host-pathogen interactions (see [2]).

Evidence for a positive relationship between parasite multiplication and virulence comes mostly from microparasites infecting animals (reviewed by [7], [8], [10]). In contrast, experimentation with plant parasites is much scarcer and often does not support a multiplication-virulence relationship, thus hampering the generalisation of the trade-off hypothesis. Overall, the few reports on plant-virus systems have mostly failed to reveal a link between virus multiplication and virulence [11]–[13] but see also [14] for exceptions). In addition, abundant circumstantial evidence (*i.e.* absence of correlation between accumulation level and symptom severity) suggest that the trade-off hypothesis would not be extendable to plant viruses. Therefore, it is highly relevant to experimentally test how widespread there is a positive relationship between within-host parasite multiplication and virulence in plant-virus systems. Evaluating the trade-off hypothesis assumptions with plant parasites is economically more feasible and involves less ethical constraints than with most animal systems. Thus, plant-parasite systems have a large potential to test the predictions of theoretical models based on the trade-off hypothesis or, more generally, to test hypotheses on the evolution of virulence and host-parasite co-evolution.

In this work we aim to study the relationship between parasite multiplication and virulence in a plant-virus system derived from the widespread pathogen *Cucumber mosaic virus* (CMV) and the crucifer *Arabidopsis thaliana* L. *Arabidopsis* has been developed as the model organism for molecular plant genetics, including the analysis of the mechanisms of resistance to parasites [15]. More recently, *Arabidopsis* has also been used in the study of fitness consequences of herbivores and parasites on plants and of plant-parasite co-evolution [16]–[18]. In this context, it has been shown that *Arabidopsis* reactions to CMV infection vary according to genotype [19]. CMV is a plant-infecting virus considered as a typical generalist parasite. It shows the broadest host range known for a plant virus, infecting about 1,200 host species in more than 100 plant families. CMV has isometric particles that encapsidate separately the genomic segments of a tri-partite messenger-sense single-stranded RNA genome with five genes. RNA1 is monocistronic, encoding for protein 1a. RNA2 encodes for protein 2a, which interacts with protein 1a in the viral RNA-dependent RNA-replicase, and protein 2b, a suppressor of the virus-induced gene silencing resistance reaction of the plant. RNA 3 encodes protein 3a, which is required for cell-to-cell movement of virus infection through the plasmodesmata, and the coat protein, also required for cell-to-cell movement, for systemic movement via the phloem and for aphid transmission. CMV is horizontally transmitted by more than 70 species of aphids in a non-persistent manner, so that the virus is acquired from an infected plant and transmitted to a new one in short periods of time (seconds to minutes), the aphid remaining viruliferous also for short periods (hours). CMV isolates are highly diverse, and have been classified in three subgroups, IA, IB and II, according to the similarity of their genomic RNA sequences. Isolates in these subgroups may also differ in host range and in the severity of symptoms induced in common hosts (for reviews see [20], [21]).

Most experimental analyses of the relationship between parasite multiplication and virulence have focussed on different parasite genotypes infecting a single host genotype. However, host-parasite interactions, including virulence, may depend on the genotypes of both host and parasite [22], [23]. Thus, to test the validity of the trade-off hypothesis assumptions for the *Arabidopsis*-CMV host-parasite system, in this report we have analysed the interaction of three CMV isolates with twenty one wild genotypes (referred to as accessions) of *Arabidopsis*. The effect of virus infection on the plant fitness, *i.e.*, virulence, was estimated from the number of viable seeds produced by infected plants relative to mock-inoculated ones. To understand the processes resulting in virulence we have quantified the multiplication of CMV and the effects of virus infection on plant growth and seed production, in the twenty one accessions of *Arabidopsis*. Overall, the results of these analyses did not provide evidence for a significant relationship between virus multiplication and virulence in this plant-virus system. These results could be explained, at least partly, by accession-specific tolerance mechanisms operating on plant growth and on resource allocation to seed production. A positive correlation between virus multiplication and virulence was found, though, for a small number of the analysed accessions. These findings indicate the need to analyse the interaction among different genotypes of host and parasite when testing the general validity of virulence evolution hypotheses.

## Results

### Variability of CMV symptoms on *Arabidopsis* accessions

Plants from twenty one accessions of *A. thaliana* selected to include the broad geographical and genetic variation of the species (Table 1 and Material and Methods) were inoculated with three CMV isolates: Fny-CMV and De72-CMV, belonging to subgroup IA of CMV isolates, and LS-CMV, belonging to subgroup II. Ten replicates (*i.e.*, plants) were done for each CMV isolate/*Arabidopsis* accession treatment. An additional mock-inoculated treatment, also with ten replicates, was included for each accession. Plants were inoculated in three rosette leaves per plant, at the emergence of inflorescences, when the first flower bud was visible (growth stage 5/5.1 as described in Boyes et al. [24]).

**Table 1:** Origin and life cycle length of *Arabidopsis thaliana* accessions.

Name	Origin	Life cycle length a)
An-1	Amberes (Belgium)	55.4±1.744
Bay-0	Bayreuth (Germany)	64.7±1.155
Boa-0	Boadilla del Monte (Spain)	78.7±2.558
Cad-0	Candelario (Spain)	84.0±0.720
Cdm-0	Caldas de Miravete (Spain)	83.0±0.001
Cen-1	Centenera (Spain)	66.6±1.298
Co-1	Coimbra (Portugal)	74.6±0.804
Col-1	Columbia (Unknown)	66.7±1.556
Cum-0	Cumbres Mayores (Spain)	72.0±1.414
Cvi	Cape Verde Islands	60.5±0.724
Fei-0	Santa María da Feira (Portugal)	65.2±2.398
Kas-0	Kashmir (India)	78.4±1.416
Kas-2	Kashmir (India)	82.1±1.328
Kyo-1	Kyoto (Japan)	67.6±2.062
L*er*	Landsberg (Poland)	61.1±0.867
Ll-0	Llagostera (Spain)	81.5±0.805
Mer-0	Mérida (Spain)	66.6±1.288
Pro-0	Proaza (Spain)	57.7±1.912
Shak	Shakdara (Tadjikistan)	44.9±1.440
Sne	Sierra Nevada (Spain)	118.5±0.003
Vif-0	Villafáfila (Spain)	125.0±0.772

a) Data are mean±standard error of days from planting to complete senescence (stage 9.7 of Boyes et al. 2001) in the assayed conditions. Plants from all accessions were planted five days after germination (see Material and Methods).

To test for the role of plant developmental stage at the start of the infection on pathogen susceptibility and virulence, a second experiment was done with the same experimental design except that only eighteen *Arabidopsis* accessions were assayed. Accessions Co-1, which developed a systemic veinal necrosis upon infection with subgroup IA CMV isolates in the first experiment, and accessions Mer-0 and Cdm-0, which failed to germinate, were not included. In this second experiment plants were inoculated at an earlier vegetative growth stage, when they had developed 4–5 rosette leaves (stages 1.04–1.05 of Boyes et al. [24]).

**Figure 1:**
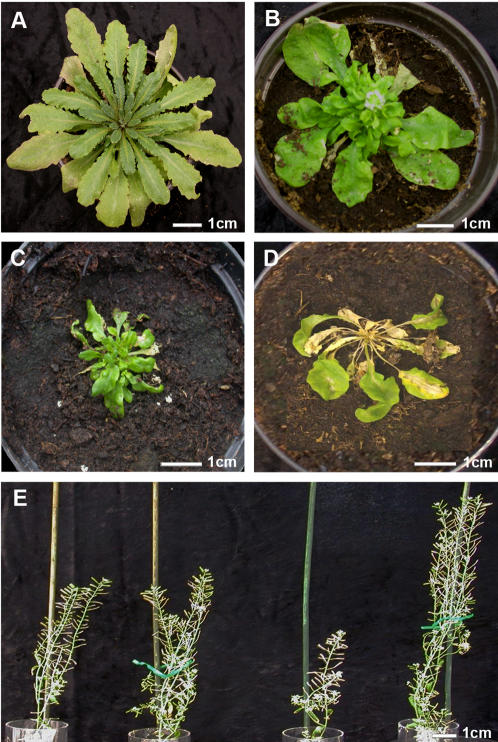
Symptoms of CMV infection in *Arabidopsis thaliana* accessions. A. Asymptomatic infection of Ll-0 by LS-CMV. B. Leaf curl and lamina reduction in rosette leaves of Boa-0 infected by LS-CMV. C. Leaf curl and severe lamina reduction in Kyo-1 plants infected by Fny-CMV. D. Systemic veinal necrosis in Co-1 infected by Fny-CMV. E. Various degrees of stunting of L*er* infected by CMV, from left to right: Fny-CMV, De72-CMV, LS-CMV, mock-inoculated control. Note the different scales in the bottom-right corner of each panel.

All assayed *Arabidopsis* accessions were susceptible to the three CMV isolates in both experiments. No immune or hypersensitive resistance responses were observed. Upon infection, plants remained asymptomatic (Fig. 1A) or developed a variety of symptoms, depending on the isolate-accession interaction. In most cases rosette leaves showed different degrees of leaf curl and lamina reduction (Fig. 1B), and plants developed different degrees of stunting, particularly noticeable in the reproductive structures (Fig. 1E). The most severe symptoms developed in plants of accessions Kyo-1 and Co-1 upon infection by subgroup IA CMV isolates. In Kyo-1 plants, stunting was extreme and inflorescences were not produced (Fig. 1C), while Co-1 plants developed a systemic veinal necrosis and also failed to flower (Fig. 1D). There was no obvious relationship between the severity of symptom expression and the length of life cycle of the accession (Table 1).

### Multiplication of CMV isolates on *Arabidopsis* accessions

**Figure 2:**
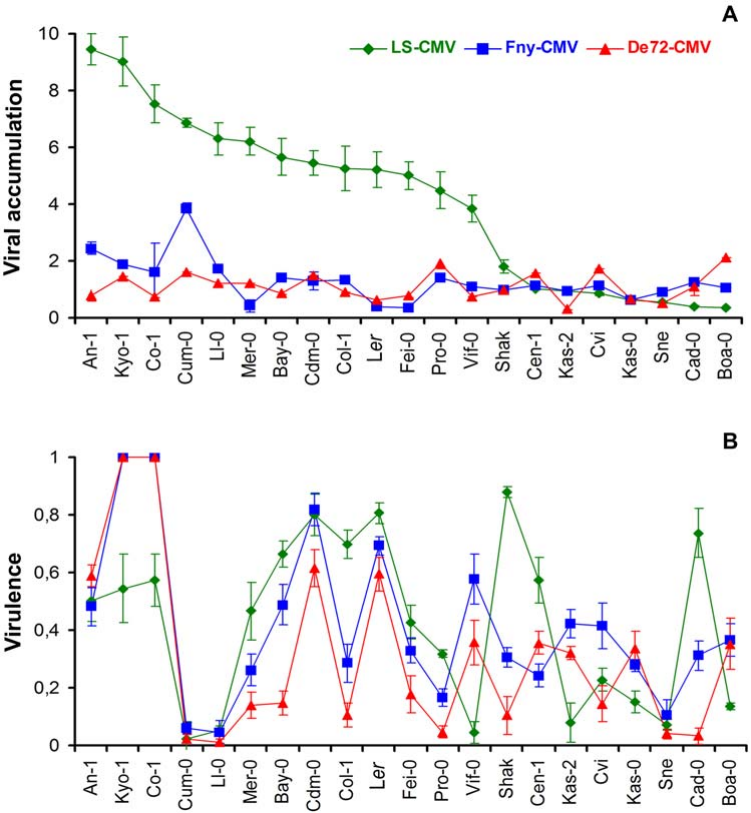
CMV accumulation and virulence on *Arabidopsis thaliana* accessions. A. Accumulation of viral RNA (µg/g fresh weight) is estimated for 1∶1 mix of inoculated and systemically infected leaves of plants inoculated at reproductive stage (Experiment 1). B. Virulence (*V*) is estimated as the effect of virus infection at reproductive stage on production of viable seeds (see text for details). Data are mean±standard errors of ten replicates. Accessions are presented according to the accumulation levels of LS-CMV in panel A. CMV isolate: LS-CMV (green lozenges), Fny-CMV (blue squares) and De72-CMV (red triangles).

CMV accumulation was quantified as the amount of viral RNA detected in leaves by blot hybridization (see statistical parameters of the frequency distributions in Table S1). Virus accumulation in plants inoculated after induction of flowering, at growth stage 5/5.1 (Experiment 1), differed significantly depending on the virus isolate, on the *Arabidopsis* accession and on the interaction between these two factors (*P*<1×10^−5^). Isolates and the interaction accession x isolate explained a larger fraction of the variance of virus accumulation than accessions (*VC* = 32.18, *VC* = 16.22, *VC* = 36.38 for isolate, accession and interaction, respectively). Accumulation of LS-CMV across the *Arabidopsis* accessions was significantly higher than that of Fny-CMV and De72-CMV, which accumulated to similar levels (Fig. 2A). Quantitative variation for virus accumulation among the different *Arabidopsis* accessions (Fig. 2A), indicated different degrees of resistance/susceptibility, defined as the ability of the host to limit/sustain the multiplication of the parasite [25].

Similar results were obtained in Experiment 2, when plants were inoculated at vegetative stage (Fig. S1A). Comparison of virus accumulation in the two experiments showed a significant interaction between accession and experiment, suggesting that virus accumulation in each genotype depends on the developmental stage at the time of inoculation.

Broad-sense heritability of virus accumulation in *Arabidopsis* plants ranged from moderate to high (*h^2^_b_* = 0.22–0.90) depending on the CMV isolate and the experiment. Therefore, there is significant genetic variation among the studied *Arabidopsis* accessions for the ability to sustain CMV multiplication, although large differences were observed depending on the viral isolate and the moment of infection. Virus accumulation and length of accession life cycle were not correlated in any experiment (*P*>0.201).

### Effect of CMV infection on *Arabidopsis* plant growth-related traits

Virulence in plant-parasite interactions has often been estimated from the effect of parasite infection on plant growth. It is reasonable to hypothesise that differences in plant architecture between the analysed *Arabidopsis* accessions, associated with differences in resource allocation to the different organs, might condition the effects of virus infection on plant growth and on the number of viable seeds produced. Thus, we analysed first the relationship between above-ground biomass (*BM*) and the weight of seeds produced per plant (*SW*) in mock-inoculated plants for all 21 accessions (see Table S1).

**Figure 3:**
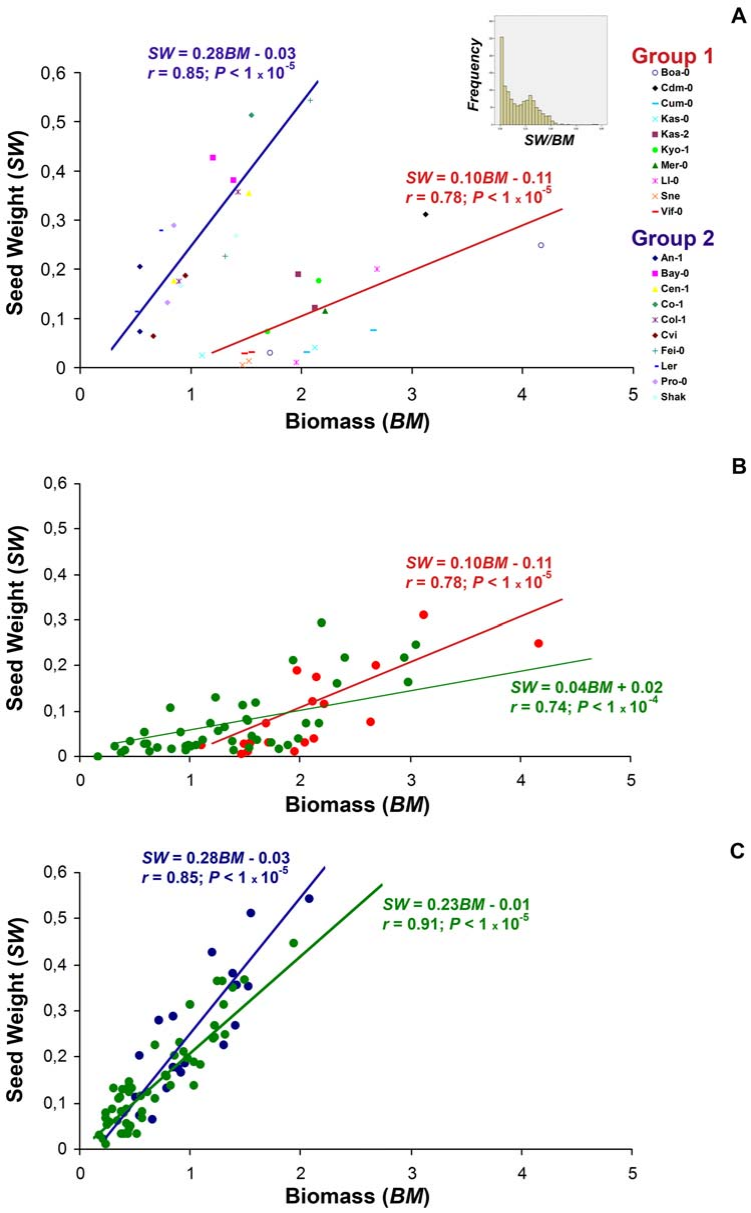
Relationship between above-ground biomass (*BM*) and the weight of seeds produced per plant (*SW*) in *Arabidopsis thaliana* accessions. A. Correlation *SW/BM* for accession average values of mock-inoculated plants of allometry group 1 (red) and allometry group 2 (blue), and frequency histogram of this relationship represented considering ten replicates of each accession. B. Effect of infection in *SW/BM* relationship (green) for allometry group 1. C. Effect of infection in *SW/BM* relationship (green) for allometry group 2, data are mean values of each accession.

In mock-inoculated plants, biomass showed a positive correlation with length of life cycle, while seed weight showed a negative correlation *(r*>0.68, *P*<0.004 for biomass, *r*>−0.62, *P*<0.011 for seed weight). In both experiments the ratio *SW/BM* showed a strong negative correlation with length of life cycle (*r*>−0.87, *P*<1×10^−4^). However no significant correlation between seed weight and biomass was found when all accessions were analysed together. The ratio *SW/BM* significantly differed among accessions (*P* = 1×10^−5^), but not between experiments, the interaction accession x experiment not being significant either (*P*≥0.100). Values of *SW/BM* varied largely and showed a bimodal distribution (Fig. 3A). Accordingly, accessions were divided into two groups with distinct *SW/BM* ratios: those with values of *SW/BM*<0.125 (group 1, mean value of 0.042±0.004), and those with *SW/BM*>0.125 (group 2, mean value 0.239±0.008). These two groups largely corresponded to accessions with life cycle longer (group 1) or shorter (group 2) than 70 days. When these two groups of accessions were analysed separately, significant and distinct linear allometric relationships were found between seed weight and biomass (Fig. 3). The significance of the regression for group 1 was largely improved when accession Cad-0 was removed from the analysis. Thus, we will consider two allometric groups of *Arabidopsis* accessions: group 1, including accessions Boa-0, Cdm-0, Cum-0, Kas-0, Kas-2, Kyo-1, Ll-0, Mer-0, Sne and Vif-0, and group 2, including accessions An-1, Bay-0, Cen-1, Co-1, Col-1, Cvi, Fei-0, L*er*, Pro-0 and Shak.

To quantify the effect of CMV infection on plant growth, the ratio of above-ground biomass in infected *vs.* mock-inoculated plants (*BM_i_*/*BM_m_*, where *i* and *m* denote infected and mock inoculated plants, respectively) was determined for each accession (see Table S1 and Fig. S2). In both experiments a negative correlation between *BM_i_*/*BM_m_* and length of life cycle was apparent (*r*>−0.56, *P*<0.008). Both, when plants were inoculated at vegetative and reproductive stage, *BM_i_*/*BM_m_* differed significantly according to virus isolate, according to accession and according to the interaction between both factors (*P*≤5×10^−4^). On average, the effect of infection on plant growth was higher for Fny-CMV and smaller when infection occurred at vegetative stage than at reproductive stage (Fig. S2). In addition, a significant accession x experiment interaction (*P* = 1×10^−5^) was detected, suggesting that the reduction of plant growth due to CMV infection depended on the time of infection for each accession. When a similar analysis was done with accessions of the allometric group 1, plants showed larger biomass reduction in experiment 2 than experiment 1 (*BM_i_*/*BM_m_* mean values of 0.47±0.02 and 0.69±0.02, respectively). However, for group 2 of accessions, differences in *BM_i_*/*BM_m_* ratio between experiments were not significant. These results suggest that the developmental stage of inoculation affected differentially the reduction of biomass in both groups of accessions. Nevertheless we cannot discard that differences between experiments might also be due to unknown environmental factors.

### Virulence of CMV on *Arabidopsis* accessions: effect of infection on seed production-related traits

If virulence is strictly defined as the effect of parasite infection on host fitness, the relevant parameter to measure it should be the number of viable seeds produced per plant. To this end, we first estimated the total number of seeds produced per plant using the relationship between seed number (*SN*) and seed weight (*SW*), which was determined from the weight of 200 seeds in five plants per treatment. This later trait differed among accessions (*P*<1×10^−5^) but was not affected by CMV infection in any of the two experiments (*P*≥0.115). Seed viability was estimated as the percentage of seed germination (*SG*) determined from five plants per treatment. For each accession, the effect of virus infection on the production of viable seeds was estimated as the ratio of seed number in infected *vs.* mock-inoculate plants multiplied by the percentage seed germination, and CMV virulence as *V = 1–(SN_i_xSG_i_)/(SN_m_xSG_m_)*. Statistical parameters of the frequency distributions of *SN*, *SW*, *SG* and *V* are shown in Table S1.

In both experiments a negative correlation between virulence and length of life cycle was found (*r*>−0.34, *P*<0.001). CMV virulence significantly differed among CMV isolates, among *Arabidopsis* accessions and due to the interaction between these two factors (*P*≤4×10^−2^) (see Fig. 2B and Fig. S1B). Accessions accounted for a larger fraction of the virulence variance than isolates or the interaction between both factors (e.g., *VC* = 3.99, *VC* = 13.85, *VC* = 3.99 for isolate, accession and interaction, respectively, when infected at reproductive stage). Virulence was on average higher for Fny-CMV than for the other two isolates. Virulence on the different accessions varied from 0 (e.g., accession Ll-0) to 1, (accession Co-1 and Kyo-1 infected with isolates Fny-CMV and De72-CMV, which failed to produced seeds), indicating different degrees of tolerance, defined as the host ability to reduce the effect of infection on its fitness [25]. Differences in virulence between experiments were only marginally significant (*P* = 0.060). Therefore, virulence depended mainly on the virus isolate and the *Arabidopsis* accession.

When accessions of group 1 or group 2 were analysed separately, virulence was significantly lower for group 1 than for group 2 (mean values of 0.18±0.04 and 0.36±0.03, respectively). In addition, a significant difference between experiments was found for group 2 (*P* = 0.04) but not for group 1 (*P* = 0.63). On average, virulence was higher in accessions of group 2 when infected at vegetative than reproductive phase (mean values of 0.39±0.04 and 0.33±0.04, respectively).

Furthermore, the effect of CMV on the *SW/BM* ratio was also analysed (Fig. 3). The mean value of the *SW/BM* ratio in accessions of group 1 was 0.04 either when infected or mock-inoculated. For accessions of group 2, the mean value of the *SW/BM* ratio was 0.19 for infected plants but 0.24 for mock-inoculated controls. The regression lines of seed weight on biomass were compared between infected and mock-inoculated plants in each allometry group, the lines of both groups of accessions differing significantly (Fig. 3B and 3C). For group 1, most infected plants showed biomass values below the intersect of the two regression lines, indicating that for these accessions the effect of virus infection on seed weight was relatively smaller than on biomass (Fig. 3B). In contrast, for the accessions of group 2 virus infection reduced seed production relatively more than plant growth (Fig. 3C).

### Relationship between virus multiplication and virulence

When the relationship between CMV multiplication and virulence (*V*) was analysed comparing all accessions and isolates together, no significant correlation was found between these traits, either when plants were infected at reproductive stage (Experiment 1) or at vegetative stage (Experiment 2) (*P* ≥ 0.25). Also, no significant correlation was found between virus multiplication and virulence when data for each CMV isolate were analysed separately (*P* ≥ 0.42).

As described above with the two allometric groups, *Arabidopsis* accessions differ in resource allocation to seed production, which conditions the effect of virus infection on seed production. Since these differences might mask possible relationships between virus multiplication and virulence, correlations were also analysed for the two allometric groups of accessions independently. Again, no significant correlation between virus accumulation and virulence was found for any of the groups (*P* ≥ 0.25).

Furthermore, we also analysed the relationship between virus multiplication and virulence in each accession separately. Five accessions (An-1, Bay-0, Col-1, Pro-0 and Shak) showed significant correlation (*P* ≤ 0.05) when infected at reproductive stage and four accessions (An-1, Cvi, L*er* and Pro-0) did so when infected at vegetative stage. All of these accessions belonged to allometry group 2, but only two of them showed a significant relationship in both experiments. The fraction of virulence variance explained by virus multiplication varied largely among the accessions (between *R^2^* = 0.03 for Pro-0 and *R^2^* = 0.55 for An-1, both infected at vegetative stage, *P*<0.008), but most cases presented *R^2^* values between 0.2 and 0.4.

Finally, CMV multiplication was not correlated with its effect on any of the two components of host fitness, *BM_i_*/*BM_m_* and S*W/BM*, when all accessions were analysed either together or separated according to allometric group (*P*>0.10).

## Discussion

We have analysed the relationship between within-host multiplication rates and virulence in the plant-virus system *Arabidopsis*-CMV. Previous work from our group had shown that rates of horizontal transmission of CMV by aphid vectors were positively correlated with levels of within host multiplication [26], one of the assumptions of the trade-off hypothesis. However, data did not support a positive association between virulence and within-host multiplication rates [11], [12]. Limitations of these analyses were that the effect of virus infection on plant growth and life span were used as correlates of virulence, rather than the effect of infection in plant fitness. Also, different viral genotypes infecting a single host genotype or different host plant species were compared, the relationship between virus multiplication and virulence across different virus and host genotypes of the same species have not been explored. To overcome these limitations and to test the general validity of the trade-off hypothesis assumptions for a plant-virus system, we have analysed the interaction of three CMV isolates with 21 *A. thaliana* accessions. This virus-host system has been chosen because CMV is a virus frequently found infecting natural populations of *Arabidopsis* (unpublished results) and it avoids the problems associated with analyses of naïve host-pathogen combinations. Ideally, CMV isolates from *Arabidopsis* should have been used in this work, but no CMV isolate from *Arabidopsis* is available at present. Hence we used CMV isolates representing the genetic variation of this virus: two well characterised isolates, Fny-CMV and LS-CMV, representing subgroups I and II of CMV isolates, and a field isolate from *Diplotaxis*. Virus multiplication rates were estimated by viral RNA accumulation in leaves, and virulence was estimated as the effect of virus infection on host relative fitness. Although theoretical models of virulence evolution usually employ a simplified measurement of virulence, defined as the increase in host mortality due to parasite infection [5], CMV, like most plant viruses is not lethal, and its virulence cannot be quantified as the instantaneous mortality rate. Quantifying virulence as morbidity (decrease in host biomass) or, more precisely, as the decrease in the host fitness is a more rigorous and meaningful definition of virulence [1]. These estimates of virulence should not affect the outcome of our analyses on the trade-off hypothesis, even if we were to use formal models [11], [27].

Virus multiplication and virulence significantly varied according to the isolate x accession interaction, in agreement with the concept that phenotypes of host-parasite interactions depend on both the host and the parasite's genotypes, a concept often overlooked in models of virulence evolution [22], [23]. Gene-for-gene-like interactions have been described for the *Arabidopsis*-CMV system [19], [28]. Here we show that quantitative traits of parasite multiplication and host damage also depend on genotype-genotype combinations, as was reported previously for the interaction of *Arabidopsis* with *Pseudomonas syringae* and *Hyaloperonospora parasitica* [17], [18]. Substantial genetic variation was found among *Arabidopsis* accessions for all analysed traits, which is illustrated by the moderate heritabilities estimated for most of them, though these differ considerably depending on the viral isolate (Table S1).

Some isolate x accession interactions led to unique phenotypes qualitatively different from the rest such as the systemic veinal necrosis observed in Co-1 accession (previously reported to have hypersensitive resistance to some CMV strains [19]) and the extreme rosette reduction with no flower production of Kyo-1, both when infected by isolates Fny-CMV and De72-CMV. For these interactions, virulence had a maximum value of 1, since plants failed to produce any seed. In these interactions characterised by unique symptoms, virus multiplication in the infected plant and virulence were unrelated. These results agree with numerous reports on strains and mutants of many plant viruses showing that symptom severity and virus multiplication were uncoupled [29]–[34]. The pathogenesis of plant viruses is poorly understood, but virus infection results in misregulation of plant genes involved in plant growth and development which may occur in non-infected as well as in infected tissues [35], [36]. It might be speculated that the highly host-virus specific effects of virus infection require different threshold levels of virus multiplication, so that a relationship between virus multiplication and virulence would not occur.

For the other 59 isolate x accession interactions, a quantitative gradation of symptom severity, virus accumulation and of the effect of virus infection in plant biomass and in viable seed production was observed. The two allometric groups of accessions distinguished in this work differ in the effects of CMV infection in two contrasting aspects. First, the effect of virus infection on plant biomass was on average lower and it seemed independent of the developmental stage at infection for accessions of group 2, while it was higher for plants of group 1 when infected at vegetative than reproductive stage. Second, virulence was on average lower and apparently independent of the plant developmental stage at infection for accessions of group 1, whereas it was higher for plants of group 2 when infected at vegetative stage. Defining tolerance as the host ability to reduce the effect of infection on its fitness [25], these results indicate the presence of tolerance mechanisms to CMV infection in *Arabidopsis.* In addition, tolerance mechanisms seem to operate partly independently at two levels, plant growth and resource allocation to seed production. Tolerance mechanisms will depend on the plant genotype, as they are related to life cycle length and/or *SW/BM* ratio, two variables strongly correlated. For accessions of group 1, seed production tolerance is as effective when plants are infected early in the vegetative stage as when they are infected at reproductive stage. Thus, accessions of group 1 were able to compensate the effect of CMV infection on biomass by allocating more resources to seed production, as shown by the regressions of *SW* on *BM* (Fig. 3B and 3C). This is not the case for accessions of group 2, where the earlier the virus infects, the more severe reduction in seed production. The behaviour of accessions of group 2 is similar to that observed in crop species where the earlier the viruses infect crops, the more severe the reduction in yield, for crops where either the vegetative or the reproductive organs are the economic parts of the plant [37]. These results suggest that domestication and breeding for life cycle, plant growth and resistance traits might have reduced genetic variation for tolerance mechanisms to viruses, in agreement with the reduced tolerance to parasites observed in crops compared to wild plants [25] and in improved varieties compared to crop land races [38].

Analysis of the relationship between virus accumulation levels and virulence for the 59 interactions showing quantitative variation for both traits did not detect any significant correlation. It has been proposed that non-linear tolerance to parasites and herbivores would result in no clear relationship between parasite multiplication and host damage [39]. Accordingly, tolerance has been shown to explain the lack of covariation between multiplication of *P. syringae* in *Arabidopsis* and its effect on host fitness [17]. Tolerance mechanisms could have also an important role in explaining our results, where no correlation between virus multiplication and virulence was found in group 1 of accessions, with effective tolerance mechanisms, or in group 2, with less effective tolerance mechanisms operating only on the effect of virus infection on plant biomass. Nevertheless, a positive relationship between virus multiplication and virulence was found for some accessions of group 2.

For animal or bacterial host systems, a positive correlation between parasite multiplication and virulence has been reported often ([8], [40]–[50], but see also [51], [52]). Most of these analyses have focused on different parasite genotypes infecting a single host genotype, and do not test for possible interactions between host and parasite genotypes in the expression of phenotypes. It is possible that, like for the *Arabidopsis*-CMV system, the reported results for some of these systems were a property of the particular host genotype and/or experimental conditions of the analysis. When different genotypes of host and parasite have been analysed simultaneously, phenotypic traits, including virulence, have been shown to depend on the interaction of host and parasite genotypes [16], [18], [53]–[56]. However, in contrast to our results, Salvaudon et al. [18] have shown for the interaction between two strains of the oomycete *H. parasitica* and seven accessions of *Arabidopsis* that the more virulent parasite strain was the most transmissible one, in concordance with the trade-off hypothesis. A relationship between parasite fitness and virulence may also be inferred from the analysis of genotype x genotype interaction reported for the plant *Silene inflata* and the fungus *Microbotryum violaceum* [53], or from the analysis of the interaction between *A. thaliana* and *Pseudomonas viridiflava* [16]. Thus, a lack of correlation between parasite multiplication and virulence does not seem to be a general property of plant-parasite systems, for which tolerance has been more often involved in interactions with natural enemies than for animals [39].

Two major conclusions can be drawn from the results reported here. First, the central assumption of the trade-off hypothesis, *i.e.*, that virulence is correlated with parasite multiplication within the infected host, does not hold for the system *A. thaliana*-CMV. The few reported analyses of plant-virus interactions mostly have failed to detect a positive correlation between virus accumulation and virulence [11]–[13], [57], [58] or between symptom severity and virus accumulation [29]–[34], although there are some notable exceptions [14]. Second, for a host-parasite system the central assumption of the trade-off hypothesis may hold only for specific combinations of parasite and host genotypes under particular environmental conditions. Further analyses of the trade-off hypothesis assumptions involving different genotypes of parasite and host in different species are required to know how general these properties are, since the relationship between parasite multiplication and virulence will condition parasite evolution, and host-parasite co-evolution.

## Materials and Methods

### Viral isolates and *Arabidopsis thaliana* accessions

Three CMV isolates were used for all experiments: Fny-CMV and De72-CMV, belonging to subgroup IA of CMV strains, and LS-CMV, belonging to subgroup II [59]. Isolate De72-CMV was obtained from a single field-infected plant of *Diplotaxis erucoides* L. (*Brassicaceae*) from Central Spain [60], and was propagated in tobacco (*Nicotiana tabacum* L. cv. Xanthi-nc) plants. Fny-CMV and LS-CMV were derived from biologically active cDNA clones [61], [62] by *in vitro* transcription with T7 RNA polymerase (New England Biolabs, Ipswich MA). Transcripts were used to infect tobacco plants for virus multiplication. CMV virions were purified from infected tobacco leaves as described in [63] and viral RNA was extracted by virion disruption with phenol and sodium dodecyl sulphate.

Twenty one accessions of *Arabidopsis thaliana* were used (Table 1). Ten accessions represented the wide Eurasian geographic distribution of the species and were kindly obtained from Maarten Koornneef (Max Planck Institute for Plant breeding, Cologne, Germany). The remaining eleven accessions represented *Arabidopsis* distribution in the Iberian Peninsula, which has been suggested as a Pleistocene glacial refuge for *Arabidopsis* [64]. Iberian accessions were obtained from Maarten Koorneef (Co-1 and Ll-0) or Carlos Alonso-Blanco (unpublished). These accessions were selected to include a broad amount of natural genetic variation of the species, based on AFLP data [64], SNP markers [65] and microsatellite (Alonso-Blanco, unpublished). All the accessions were multiplied simultaneously in the same greenhouse to obtain the seeds used for the experiments described in this work. Hence, maternal effects are not expected and are not further considered. For plant growth, seeds of each accession were sown on filter paper soaked with water in a single plastic Petri dishes, and stratified in darkness at 4°C for 3 days before transferring for germination to a growth chamber (22°C, 14 h light and 70% relative humidity). Five day-old seedlings were planted in soil containing pots 10.5 cm of diameter, 0.43 l volume and grown in a greenhouse (25/20°C day/night, 16 h light). In each experiment, 10 individual plants were grown per accession in a completely randomised greenhouse design.

*Arabidopsis* accessions differ in the duration of their post-germination life cycle, which was measured as the mean number of days from planting till post-reproductive final senescence (Table 1).

Plants were mechanically inoculated with purified CMV RNA (100 ng/µl) in 0.1 M Na_2_HPO_4_.

### Quantification of CMV accumulation

CMV accumulation was quantified as viral RNA accumulation. Total nucleic acid extracts from 0.01 g (fresh weight) of leaves were obtained using TRI-reagent (Sigma-Aldrich, St. Louis, Missouri, USA). RNA quantification was done by dot-blot hybridization with ^32^P-labeled RNA probes specific for CMV, and hybridization signal was further analyzed by densitometry [66]. In each blot, internal CMV standards for subgroup IA (Fny-CMV or De72-CMV) and subgroup II (LS-CMV) were included as a two-fold dilution series of purified RNA (0.5 to 0.001 µg) in nucleic acid extracts from non-inoculated *Arabidopsis* plants. Mock-inoculated samples were included as negative controls. RNA extracts from infected plants were blotted at different dilutions to ensure that hybridization signal was on the linear portion of the RNA concentration-hybridization signal curve. ^32^P-labeled RNA probes were obtained by transcription from cDNA clones representing the 3′ untranslated region of the three genomic RNAs, which is highly homologous within a CMV isolate. For subgroup I isolates, Fny-CMV and De72-CMV, a probe representing nucleotides 1933 to 2215 of Fny-CMV RNA3 (GeneBank Acc. No. D10538) was used, for LS-CMV the probe represented nucleotides 1861 to 2193 of LS-CMV RNA3 (Acc. No AF127976). All hybridizations were done at 65°C overnight in 6× SSC, 5× Denhardt's mixture, 0.1% sodium dodecyl sulphate, and yeast tRNA at 50 µg/ml [67]. RNA hybridization signal was detected using a Typhoon 9400 scanner (GE Healthcare, Chalfont St. Giles, UK) after exposure of the Eu^+2^ store phosphor screens to the labelled samples. CMV quantification was done by densitometry analysis using ImageQuant 5.2 (Molecular Dynamics, GE Healthcare). As loading controls, parallel membranes were hybridised with a cDNA probe approximately 800 nucleotides long, complementary to barley 18 s rRNA [68], which showed no significant difference in the amount of rRNA in extracts from equal fresh weights of leaves, among infected or non-inoculated plants. In Experiment 1, inoculated at reproductive stage, virus accumulation was quantified on a 1∶1 (weight) mixture of inoculated and upper non-inoculated leaves harvested 15 days after inoculation. In Experiment 2 inoculated at an early vegetative stage, virus accumulation was quantified on a 1∶1 (weight) mixture of inoculated and upper non-inoculated leaves harvested 7 and 15 days post-inoculation, respectively.

### Estimation of virulence

Dry weight was measured at complete senescence of plants after incubation at 65°C until constant weight. Rosettes, inflorescences, and seeds were weighted together as above ground biomass (*BM*). Seeds were also weighted separately after threshing and recorded as seed weight (*SW*). The number of seeds produced per plant (*SN*) was estimated from *SW* after determining the weight of 200 seeds per plant. To quantify the effect of CMV infection on these plant growth traits the variable value of each infected plant was divided by the mean value of the mock-inoculated plants.

Seed viability was measured as the germination percentage of 200 seeds per plant (*SG*). Germination assays were done at least 60 days after harvesting to avoid differences on seed dormancy. Relative differences in seed viability between treatments and controls were determined as *SG_i_/SG_m_*, were *SG_i_* is germination percentage of seeds from infected plants and *SG_m_* is germination percentage of seeds from mock-inoculated controls. Virulence (*V)* was estimated from the reciprocal of the ratio between infected and mock-inoculated plants of the number of viable seeds produced per plant: *V = 1–(SN_i_xSG_i_)/(SN_m_xSG_m_)*, where *i* and *m* denote infected and mock-inoculated plants, respectively.

### Statistical analyses

Data on total biomass, seed weight, seed number, seed germination and their various transformations, including virulence, were homocedastic and were analysed using ANOVA. Data on virus accumulation showed heterogeneity of variances and therefore differences in viral accumulation according to CMV isolate or to *Arabidopsis* accession were also analysed by Kruskal-Wallis tests. Since ANOVA comparisons of these data led to similar results and conclusions than Kruskal-Wallis tests, for simplicity, only ANOVA analyses are shown.

Differences in viral accumulation, above-ground biomass and virulence according to CMV isolate or to *Arabidopsis* accession within each experiment were analysed by two-way ANOVA using accession and isolate as factors in a complete model. To determine if there are differences between the two experiments for these variables, a complete three-way ANOVA model was used including accession, isolate and experiment as factors. Divergence between experiments was likely due to the distinct developmental stage during inoculation but the influence of other unknown factors differing in both experimental conditions cannot be discarded. To determine whether values of analysed traits were significantly different among classes within each factor Least Significant Difference analyses were employed. Accession, isolate and experiment were considered as random effect factors in all ANOVAs. For each trait, the proportion of total variance explained by each factor was calculated as percentage by variance component (*VC*) analyses in the corresponding models described above.

Broad-sense heritability (*h^2^_b_*) of the analysed traits was estimated as the percentage of the total variance accounted by genetic (accession) differences (*h^2^_b_*_ = _σ^2^_G_/σ^2^_P_, were σ^2^_G_ is the genetic variance component of σ^2^_P_ total phenotypic variance). On all traits, σ^2^_P_ and σ^2^_G_ were derived by variance components analysis using separated univariate analyses for each viral isolate (plant heritabilities). Coefficients of genetic variation were estimated as 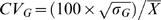, where *X̅* is the trait mean of accessions.

Correlations between variables were tested using Pearson coefficients. *SW* to *BM* linear regression equations were compared using ANOVA to test equality of slopes and intercepts [69]. All statistical analyses were performed using the statistical software package SPSS 13.0 (SPSS Inc., Chicago, USA).

## Supporting Information

Table S1 Statistical parameters of virus multiplication and virus effects on plant growth and virulence traits.
(0.07 MB DOC)

Figure S1 CMV accumulation and virulence on *Arabidopsis thaliana* accessions. A. Accumulation of viral RNA (µg/g fresh weight) is estimated for 1∶1 mix of inoculated and systemically infected leaves of plants inoculated at vegetative stage (Experiment 2). B. Virulence (*V*) is estimated as the effect of virus infection at vegetative stage on production of viable seeds (see text for details). Data are mean±standard errors of ten replicates. Accessions are presented according to the accumulation levels of LS-CMV in Fig. 2A. CMV isolate: LS-CMV (green lozenges), Fny-CMV (blue squares) and De72-CMV (red triangles).
(3.06 MB TIF)

Figure S2 Effect of viral infection on the biomass of *Arabidopsis thaliana* accessions. The effect of infection is shown for LS-CMV (green lozenges), Fny-CMV (blue squares) and De72-CMV (red triangles) for experiment 1 (A) and for experiment 2 (B). Biomass of infected plants is estimated relative to biomass of mock-inoculated controls (biomass ratio: *BMi/BMm*, where *i* and *m* denote infected and mock-inoculated plants, respectively). Data are mean±standard errors of ten replicates. Accessions are presented in the same order as in Fig. 2A.
(1.60 MB TIF)

## References

[1] Read AF (1994). The evolution of virulence.. Trends Microbiol.

[2] Bull JJ (1994). Perspective Virulence.. Evolution.

[3] Ebert D, Hamilton WD (1996). Sex against virulence: the coevolution of parasitic diseases.. Trends Ecol Evol.

[4] Frank SA (1996). Models of parasite virulence.. Q Rev Biol.

[5] Lenski RE, May RM (1994). The evolution of virulence in parasites and pathogens: reconciliation between two competing hypotheses.. J Theor Biol.

[6] Anderson RM, May R (1982). Coevolution of hosts and parasites.. Parasitology.

[7] Ebert D, Bull JJ (2003). Challenging the trade-off model for the evolution of virulence: is virulence management feasible?. Trends Microbiol.

[8] Lipsitch M, Moxon R (1997). Virulence and transmissibility of pathogens: what is the relationship?. Trends Microbiol.

[9] Restif O, Koella JC (2004). Concurrent evolution of resistance and tolerance to pathogens.. Am Nat.

[10] Ebert D (1998). Experimental evolution of parasites.. Science.

[11] Escriu F, Fraile A, García-Arenal F (2003). The evolution of virulence in a plant virus.. Evolution.

[12] Sacristán S, Fraile A, Malpica JM, García-Arenal F (2005). An analysis of host adaptation and its relationship with virulence in *Cucumber mosaic virus*.. Phytopathology.

[13] Stewart AD, Logsdon JM, Kelley SE (2005). An empirical study of the evolution of virulence under both horizontal and vertical transmission.. Evolution.

[14] Martin DP, van der Walt E, Posada D, Rybicki EP (2005). The evolutionary value of recombination is constrained by genome modularity.. PLoS Genet.

[15] Somerville C, Koornneef M (2002). A fortunate choice: the history of *Arabidopsis* as a model plant.. Nat Rev Genet.

[16] Goss EM, Bergelson J (2006). Variation in resistance and virulence in the interaction between *Arabidopsis thaliana* and a bacterial pathogen.. Evolution.

[17] Kover PX, Schaal BA (2002). Genetic variation for disease resistance and tolerance among *Arabidopsis thaliana* accessions.. Proc Natl Acad Sci USA.

[18] Salvaudon L, Héraudet V, Shykoff J (2005). Parasite-host fitness trade-offs change with parasite identity: genotype-specific interactions in a plant-pathogen system.. Evolution.

[19] Takahashi H, Goto N, Ehara Y (1994). Hypersensitive response in *Cucumber mosaic virus*-inoculated *Arabidopsis thaliana*.. Plant J.

[20] Palukaitis P, Roossinck MJ, Dietzgen RG, Francki RIB (1992). *Cucumber Mosaic Virus*.. Adv Virus Res.

[21] Palukaitis P, García-Arenal F (2003). Cucumoviruses.. Adv Virus Res.

[22] Lambrechts l, Fellous S, Koella J (2006). Coevolutionary interactions between host and parasite genotypes.. Trends Parasitol.

[23] Restif O, Koella JC (2003). Shared control of epidemiological traits in a coevolutionary model of host-parasite interactions.. Am Nat.

[24] Boyes DC, Zayed AM, Ascenzi R, McCaskill AJ, Hoffman NE (2001). Growth stage-based phenotypic analysis of *Arabidopsis*: A model for high throughput functional genomics in plants.. Plant Cell.

[25] Clarke DD (1986). Tolerance of parasites and disease in plants and its significance in host-parasite interactions.. Adv Plant Pathol.

[26] Escriu F, Perry KL, García-Arenal F (2000b). Transmissibility of *Cucumber mosaic virus* by *Aphis gossypii* correlates with viral accumulation and is affected by the presence of its satellite RNA.. Phytopathology.

[27] Day T (2002). On the evolution of virulence and the relationship between various measure of mortality.. Proc R Soc Lond B.

[28] Takahashi H, Miller J, Nozaki Y, Sukamto, Takeda M (2002). *RCY1*, an *Arabidopsis thaliana RPP8/HRT* family resistance gene, conferring resistance to *Cucumber mosaic virus* requires salicylic acid, ethylene and a novel signal transduction mechanism.. Plant J.

[29] Ding XS, Liu J, Cheng N-H, Folimonov A, Hou Y-M (2004). The *Tobacco mosaic virus* 126-kDa protein associated with virus replication and movement suppresses RNA silencing.. Mol Plant-Microbe Interact.

[30] Gal-On A (2007). *Zucchini yellow mosaic virus*: insect transmission and pathogenicity-the tails of two proteins.. Mol Plant Pathol.

[31] Handford MG, Carr JP (2007). A defect in carbohydrate metabolism ameliorates syptom severity in virus-infected *Arabidopsis thaliana*.. J Gen Virol.

[32] Rodriguez-Cerezo E, Klein PG, Shaw GJ (1991). A determinant of disease symptom severity is located in the 3′-terminal noncoding region of the RNA of a plant virus.. Proc Natl Acad Sci USA.

[33] Shi B-J, Palukaitis P, Symons RH (2002). Differential virulence by strains of *Cucumber mosaic virus* is mediated by the *2b* gene.. Mol Plant-Microbe Int.

[34] Yoon JY, Ahn HI, Kim M, Tsuda S, Ryu KH (2006). Pepper mild mottle virus pathogenicity determinants and cross protection effect of attenuated mutants in pepper.. Virus Res.

[35] Dunoyer P, Voinnet O (2005). The complex interplay between plant viruses and host RNA-silencing pathways.. Curr Opin Plant Biol.

[36] Whitham SA, Yang C, Gooding MM (2006). Global impact: Elucidating plant responses to viral infection.. Mol Plant-Microbe Int.

[37] Matthews REF (1991). Plant Virology. 3rd edition.

[38] Schafer JF (1971). Tolerance to plant disease.. Annu Rev Phytopathol.

[39] Miller MR, White A, Boots M (2006). The evolution of parasites in response to tolerance in their hosts: the good, the bad and the apparent commensalisms.. Evolution.

[40] Bull JJ, Molineux IJ (1992). Molecular genetics of adaptation in an experimental model of cooperation.. Evolution.

[41] Ebert D, Mangin KL (1997). The influence of host demography on the evolution of virulence of a microsporidian gut parasite.. Evolution.

[42] Fenner F, Ratcliffe FN (1965). Mixomatosis..

[43] Jaenike J (1996). Suboptimal virulence of an insect-parasitic nematode.. Evolution.

[44] Jakel T, Scharpfenecker M, Jitrawang P, Ruckle J, Kliemt D (2001). Reduction of transmission stages concomitant with increased host immune responses to hypervirulent *Sarcocystis singaporensis*, and natural selection for intermediate virulence.. Int J Parasitol.

[45] Jensen KH, Little T, Skorping A, Ebert D (2006). Empirical support for optimal virulence in a castrating parasite.. PLoS Biol.

[46] Mackinnon MJ, Read AF (2004). Virulence in malaria: an evolutionary viewpoint.. Phil Trans R Soc Lond B.

[47] Messenger SL, Molineux IJ, Bull JJ (1999). Virulence evolution in a virus obeys a trade-off.. Proc R Soc Lond B.

[48] Paul REL, Lafond T, Müller-Graf CDM, Nithiuthai S, Brey PT (2004). Experimental evaluation of the relationship between lethal or non-lethal virulence and transmission success in malaria parasite infections.. BMC Evol Biol.

[49] Rahme LG, Stevens EJ, Wolfort SF, Shao J, Tompkins RG (1995). Common virulence factors for bacterial pathogenicity in plants and animals.. Science.

[50] Wang I-N (2006). Lysis timing and bacteriophage fitness.. Genetics.

[51] Davies CM, Webster JP, Woolhouse MJ (2001). Trade-offs in the evolution of virulence in an indirectly transmitted macroparasite.. Proc R Soc Lond B.

[52] Levin BR, Bull JJ (1994). Short-sighted evolution and the virulence of pathogenic microorganisms.. Trends Microbiol.

[53] Kaltz O, Shykoff JA (2002). Within-and among-population variation in infectivity, latency and spore production in a host-pathogen system.. J Evol Biol.

[54] Little TK, Watt K, Ebert D (2006). Parasite-host specificity: experimental studies on the basis of parasite adaptation.. Evolution.

[55] Peever TL, Liu Y-C, Cortesi P, Milgroom MG (2000). Variation in tolerance and virulence in the chestnut blight fungus-hypovirus interaction.. Appl Environ Microbiol.

[56] Webster JP, Gower CM, Blair L (2004). Do hosts and parasites coevolve? Empirical support from the Schistosoma system.. Am Nat.

[57] Carr DE, Murphy JF, Eubanks MD (2003). The susceptibility and response of inbred and outbred *Mimulus guttatus* to infection by *Cucumber mosaic virus*.. Evol Ecol.

[58] Carr DE, Murphy JF, Eubanks MD (2006). Genetic variation and covariation for resistance and tolerance to *Cucumber mosaic virus* in *Mimulus guttatus* (Phrymaceae): a test for costs and constraints.. Heredity.

[59] Palukaitis P, García-Arenal F (2003). *Cucumber mosaic virus*. N°400.. Description of Plant Viruses.

[60] Bonnet J, Fraile A, Sacristán S, Malpica JM, García-Arenal F (2005). Role of recombination in the evolution of natural populations of *Cucumber mosaic virus*, a tripartite RNA plant virus.. Virology.

[61] Rizzo TM, Palukaitis P (1990). Construction of full-length cDNA of *Cucumber mosaic virus* RNAs 1, 2 and 3: generation of infectious RNA transcripts.. Mol Gen Genet.

[62] Zhang L, Hanada K, Palukaitis P (1994). Mapping local and systemic symptom determinants of cucumber mosaic cucumovirus in tobacco.. J Gen Virol.

[63] Lot H, Marrou J, Quiot JB, Esvan C (1972). Contribution à l'étude du virus de la mosaïque du concombre (CMV). Méthode de purification rapide du virus.. Ann Phytopathol.

[64] Sharbel TF, Haubold B, Mitchell-Olds T (2000). Genetic isolation by distance in *Arabidopsis thaliana*: biogeography and postglacial colonization of Europe.. Mol Ecol.

[65] Nordborg M, Hu TT, Ishino Y, Jhaveri J, Toomajian C (2005). The pattern of polymorphism in *Arabidopsis thaliana*.. PLoS Biol.

[66] Escriu F, Fraile A, García-Arenal F (2000a). Evolution of virulence in natural populations of the satellite RNA of *Cucumber mosaic virus*.. Phytopathology.

[67] Sambrook J, Russell DW (2001). Molecular cloning: A laboratory manual. 3rd edition.

[68] Gerlach WL, Bedbrook JR (1979). Cloning and characterization of ribosomal RNA genes from wheat and barley.. Nucleic Acids Res.

[69] Sokal RR, Rohlf FJ (1995). Biometry. 3rd edition.

