# Supplementary material for: The Relationship of Within-Host Multiplication and Virulence in a Plant-Virus System
Source: PLoS One. 2007 Aug 29;2(8):e786. doi: 10.1371/journal.pone.0000786 (PMC1950075; doi:10.1371/journal.pone.0000786)
Supplement: Table S1 — Statistical parameters of virus multiplication and virus effects on plant growth and virulence traits. (0.07 MB DOC) [file pone.0000786.s001.doc]

**Table S1.** Statistical parameters of virus multiplication and virus effects on plant growth and virulence traits.

|  |  | **Isolate^5^** | ***VA*^6^** | *BM^6^* | *BM_i_/BM_m_^6^* | *SW^6^* | *SW/BM^6^* | ***SN*^6^** | ***V*^6^** |
| --- | --- | --- | --- | --- | --- | --- | --- | --- | --- |
| **EXP. 1** |  |  |  |  |  |  |  |  |  |
|  | **Acc. Mean^1^** | ***Mock*** | - | 1.65 ± 0.06 | - | 0.23 ± 0.03 | 0.17 ± 0.01 | 10493.50 ± 484.62 | - |
|  |  | ***Fny*** | 1.30 ± 0.08 | 1.10 ± 0.09 | 0.69 ± 0.05 | 0.14 ± 0.00 | 0.15 ± 0.02 | 6296.65 ± 475.60 | 0.34 ± 0.05 |
|  |  | ***De72*** | 1.48 ± 0.07 | 1.41 ± 0.06 | 0.91 ± 0.05 | 0.17 ± 0.00 | 0.14 ± 0.01 | 7216.17 ± 466.26 | 0.20 ± 0.05 |
|  |  | ***LS*** | 4.13 ± 0.08 | 1.17 ± 0.04 | 0.69 ± 0.05 | 0.13 ± 0.00 | 0.15 ± 0.02 | 6128.97 ± 460.43 | 0.31 ± 0.05 |
|  | **Acc. Min-Max^2^** | ***Mock*** | - | 0.53 - 4.16 | - | 0.03 - 0.54 | 0.01 - 0.39 | 1037.71 - 23299.52 | - |
|  |  | ***Fny*** | 0.36 - 3.85 | 0.21 - 2.43 | 0.14 - 0.95 | 0.00 - 0.37 | 0.00 - 0.33 | 0.00 - 15850.35 | 0.02 - 1 |
|  |  | ***De72*** | 0.30 - 2.10 | 0.21 - 3.17 | 0.21 - 1.23 | 0.00 - 0.45 | 0.00 - 0.33 | 0.00 - 20733.56 | 0.01 - 1 |
|  |  | ***LS*** | 0.35 - 9.02 | 0.24 - 2.95 | 0.33 - 1.13 | 0.01 - 0.31 | 0.01 - 0.34 | 376.39 - 13996.92 | 0.01 - 0.88 |
|  | ***CV_G_*^3^** | ***Mock*** | - | 57 | - | 63 | 81 | 61 | - |
|  |  | ***Fny*** | 55 | 61 | 65 | 73 | 71 | 75 | 72 |
|  |  | ***De72*** | 09 | 63 | 52 | 71 | 87 | 72 | 94 |
|  |  | ***LS*** | 71 | 66 | 56 | 52 | 71 | 51 | 100 |
|  | **Acc. LSD^4^** | ***Mock*** | - | 0.46 | - | 0.08 | 0.04 | 5715.94 | - |
|  |  | ***Fny*** | 0.68 | 0.38 | 0.23 | 0.07 | 0.06 | 3478.98 | 0.37 |
|  |  | ***De72*** | 0.34 | 0.46 | 0.23 | 0.05 | 0.04 | 2510.63 | 0.29 |
|  |  | ***LS*** | 1.46 | 0.51 | 0.29 | 0.11 | 0.07 | 5058.41 | 0.37 |
|  | ***h^2^_b_*** | ***Mock*** | - | 0.76 | - | 0.74 | 0.89 | 0.49 | - |
|  |  | ***Fny*** | 0.46 | 0.71 | 0.75 | 0.62 | 0.72 | 0.59 | 0.29 |
|  |  | ***De72*** | 0.90 | 0.75 | 0.71 | 0.79 | 0.89 | 0.77 | 0.23 |
|  |  | ***LS*** | 0.76 | 0.64 | 0.58 | 0.22 | 0.65 | 0.23 | 0.32 |
| **EXP. 2** |  |  |  |  |  |  |  |  |  |
|  | **Acc. Mean^1^** | ***Mock*** | - | 1.32 ± 0.15 | - | 0.13 ± 0.03 | 0.12 ± 0.02 | 5815.56 ± 121.42 | - |
|  |  | ***Fny*** | 0.97 ± 0.10 | 0.45 ± 0.04 | 0.39 ± 0.05 | 0.06 ± 0.00 | 0.12 ± 0.01 | 2810.25 ± 178.88 | 0.41 ± 0.05 |
|  |  | ***De72*** | 0.13 ± 0.11 | 1.01 ± 0.08 | 0.78 ± 0.05 | 0.10 ± 0.00 | 0.11 ± 0.01 | 4371.87 ± 172.40 | 0.16 ± 0.05 |
|  |  | ***LS*** | 1.97 ± 0.12 | 0.72 ± 0.12 | 0.55 ± 0.05 | 0.08 ± 0.00 | 0.11 ± 0.01 | 3663.84 ± 175.85 | 0.38 ± 0.05 |
|  | **Acc. Min-Max^2^** | ***Mock*** | - | 0.50 - 2.50 | - | 0.01 - 0.38 | 0.01 - 0.28 | 618.89 - 15553.5 | - |
|  |  | ***Fny*** | 0.47 - 1.40 | 0.21 - 0.97 | 0.04 - 1.07 | 0.00 - 0.20 | 0.00 - 0.23 | 0.00 - 9238.18 | 0.01 - 1 |
|  |  | ***De72*** | 0.05 - 0.28 | 0.37 - 2.06 | 0.04 - 1.13 | 0.00 - 0.35 | 0.00 - 0.26 | 0.00 - 13808.28 | 0.00 - 1 |
|  |  | ***LS*** | 0.42 - 10.26 | 0.17 - 1.61 | 0.15 - 0.93 | 0.00 - 0.37 | 0.01 - 0.28 | 1082.86 -16638.21 | 0.00 - 0.96 |
|  | ***CV_G_*^3^** | ***Mock*** | - | 43 | - | 83 | 74 | 79 | - |
|  |  | ***Fny*** | 39 | 45 | 57 | 110 | 73 | 108 | 72 |
|  |  | ***De72*** | 43 | 53 | 31 | 100 | 76 | 91 | 163 |
|  |  | ***LS*** | 114 | 60 | 34 | 119 | 77 | 117 | 90 |
|  | **Acc. LSD^4^** | ***Mock*** | - | 0.23 | - | 0.08 | 0.03 | 1216.31 | - |
|  |  | ***Fny*** | 0.39 | 0.27 | 0.19 | 0.04 | 0.04 | 1608.34 | 0.33 |
|  |  | ***De72*** | 0.09 | 0.25 | 0.20 | 0.05 | 0.04 | 2073.10 | 0.40 |
|  |  | ***LS*** | 0.53 | 0.25 | 0.21 | 0.04 | 0.04 | 1616.65 | 0.36 |
|  | ***h^2^_b_*** | ***Mock*** | - | 0.74 | - | 0.73 | 0.79 | 0.74 | - |
|  |  | ***Fny*** | 0.43 | 0.31 | 0.51 | 0.71 | 0.77 | 0.73 | 0.29 |
|  |  | ***De72*** | 0.22 | 0.78 | 0.52 | 0.75 | 0.80 | 0.74 | 0.24 |
|  |  | ***LS*** | 0.44 | 0.69 | 0.39 | 0.84 | 0.79 | 0.85 | 0.24 |

**^1^** Mean value of each trait across accessions. Data are trait mean ± standard error.

**^2^** Minimum and maximum mean values across accessions.

**^3^** Coefficient of genetic variation, estimated as $CV_{G}=\left(100\times\sqrt{\sigma_{G}}\right)/\overline{X}$ where $\bar{X}$ is the trait mean of accessions.

**^4^** Least Significant Difference values.

**^5^ *Mock***: Mock-inoculated plants; ***Fny:*** Plants infected by Fny-CMV; ***De72:*** Plants infected by De72-CMV; ***LS:*** Plants infected by LS-CMV.

**^6^** ***VA***: Viral accumulation; ***BM***: Biomass; ***BM_i_/BM_m_***: Biomass ratio; ***SW***: Seed Weight; ***SW/BM***: Seed Weight to Biomass ratio; ***SN***: Seed Number; ***V***: Virulence.
