# Supplementary figures and images for: The Relationship of Within-Host Multiplication and Virulence in a Plant-Virus System
Source: PLoS One. 2007 Aug 29;2(8):e786. doi: 10.1371/journal.pone.0000786 (PMC1950075; doi:10.1371/journal.pone.0000786)

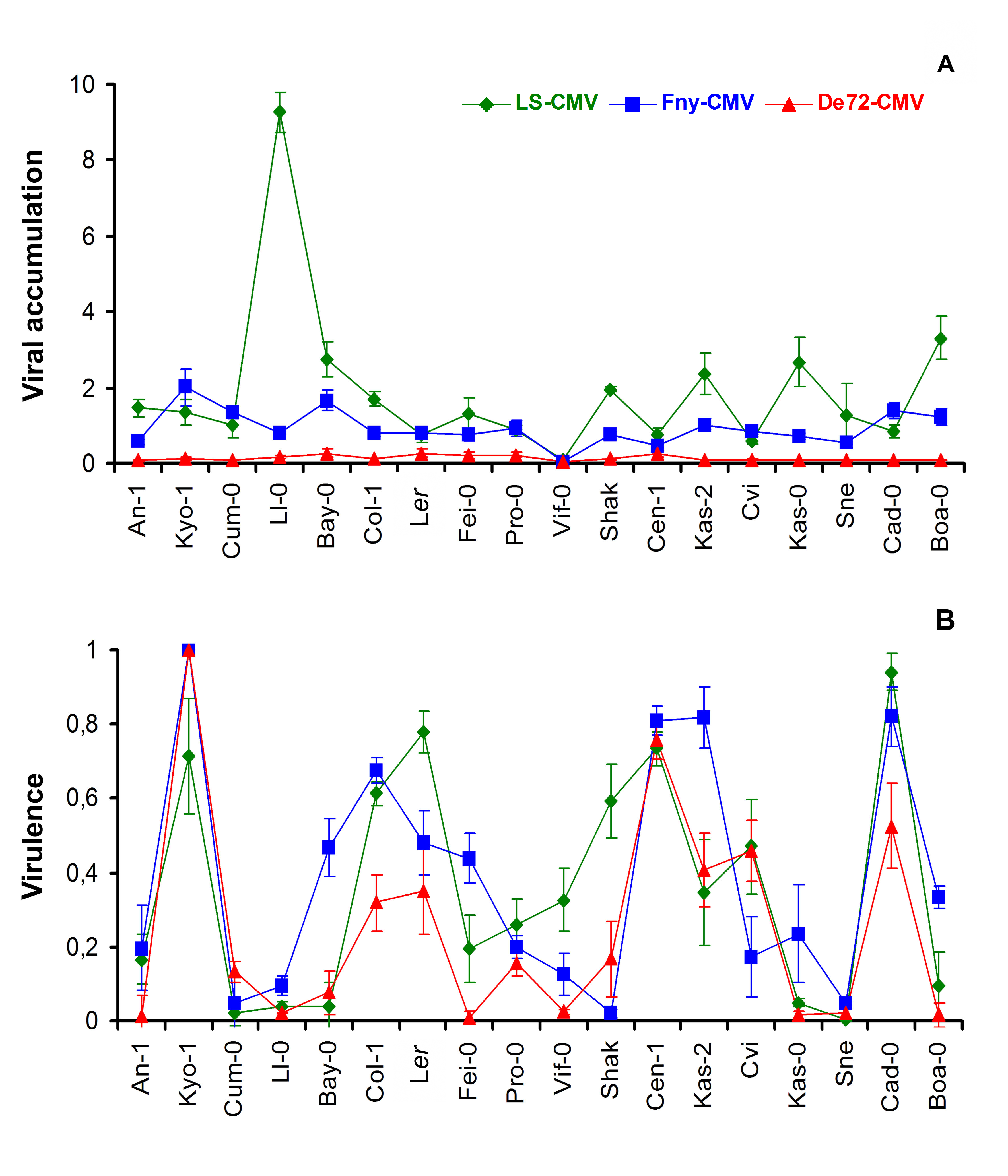

Supplement: Figure S1 — CMV accumulation and virulence on Arabidopsis thaliana accessions. A. Accumulation of viral RNA (µg/g fresh weight) is estimated for 1∶1 mix of inoculated and systemically infected leaves of plants inoculated at vegetative stage (Experiment 2). B. Virulence (V) is estimated as the effect of virus infection at vegetative stage on production of viable seeds (see text for details). Data are mean±standard errors of ten replicates. Accessions are presented according to the accumulation levels of LS-CMV in Fig. 2A. CMV isolate: LS-CMV (green lozenges), Fny-CMV (blue squares) and De72-CMV (red triangles). (3.06 MB TIF) [file pone.0000786.s002.tif]

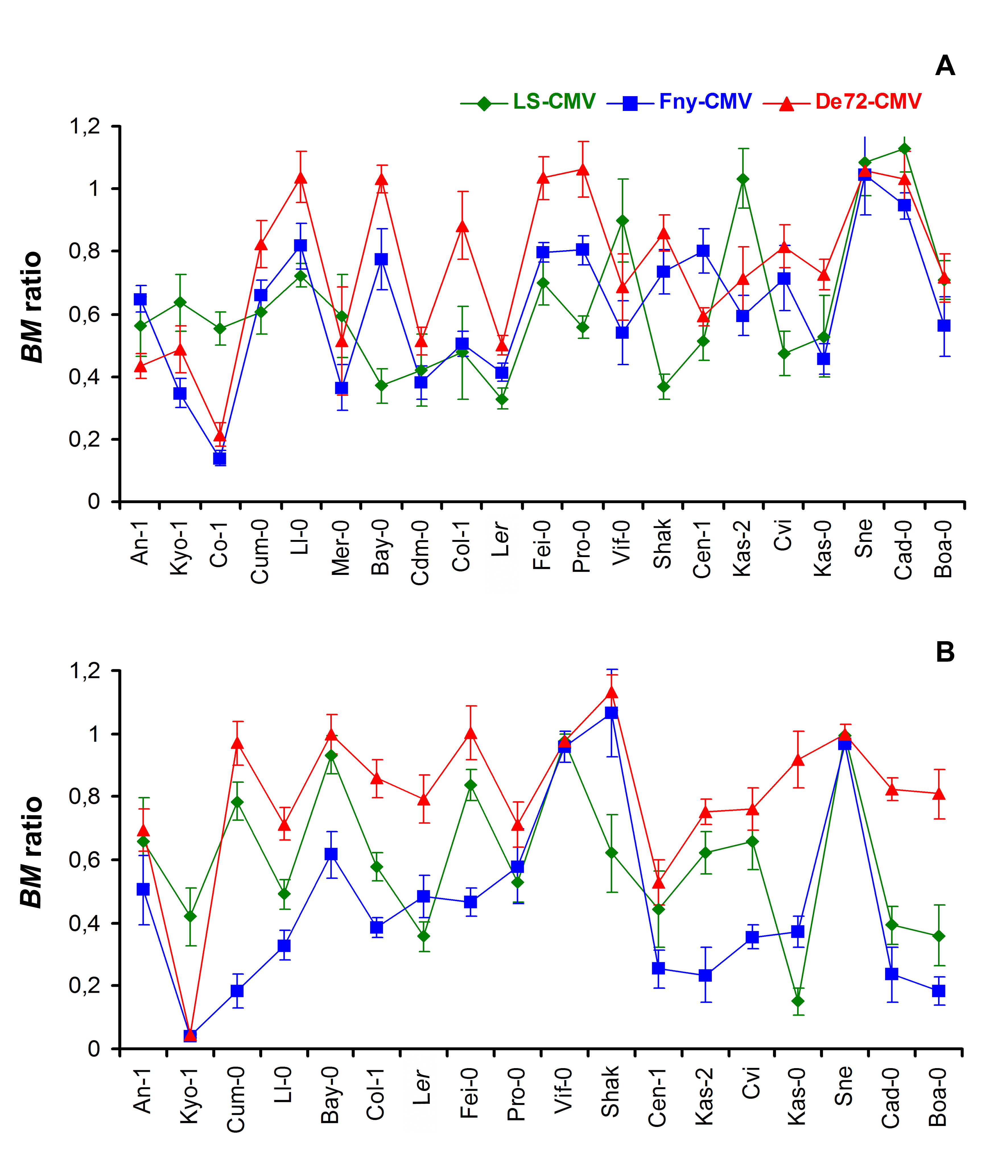

Supplement: Figure S2 — Effect of viral infection on the biomass of Arabidopsis thaliana accessions. The effect of infection is shown for LS-CMV (green lozenges), Fny-CMV (blue squares) and De72-CMV (red triangles) for experiment 1 (A) and for experiment 2 (B). Biomass of infected plants is estimated relative to biomass of mock-inoculated controls (biomass ratio: BMi/BMm, where i and m denote infected and mock-inoculated plants, respectively). Data are mean±standard errors of ten replicates. Accessions are presented in the same order as in Fig. 2A. (1.60 MB TIF) [file pone.0000786.s003.tif]
